# Mucocele-Like Lesion of the Breast

**DOI:** 10.7759/cureus.37829

**Published:** 2023-04-19

**Authors:** Brittany Q Dang, Brittany Miles, Peter Young, Jing He, Quan D Nguyen

**Affiliations:** 1 Radiology, University of Texas Medical Branch, Galveston, USA; 2 Pathology, University of Texas Medical Branch, Galveston, USA; 3 Radiology, Baylor College of Medicine, Houston, USA

**Keywords:** breast mucocele, mucocele-tumor of the breast, breast pathology, mucocele-like lesion, mucocele

## Abstract

Mucocele-like lesions (MLLs) of the breast are rare neoplasms characterized by dilated, mucin-filled epithelial ducts or cysts that can rupture and expel their contents into the surrounding stroma. They are frequently associated with atypia, dysplastic change, and, more recently, pre-malignant and malignant conditions like atypical ductal hyperplasia, ductal carcinoma, invasive carcinoma, or mucinous carcinoma. The malignant potential of MLLs is often challenging to determine from the initial histologic evaluation of a core-needle biopsy due to copious mucin and low cellularity. Therefore, at initial presentation, MLLs should be surgically excised and thoroughly evaluated for malignancy. In this paper, we present a rare case of an MLL and explore the radiology, histology, carcinogenic potential, diagnostic evaluation, and suggested management of the condition.

## Introduction

Mucocele-like lesions (MLL) of the breast are a rare breast condition that comprises <1% of benign breast biopsies [1]. Most cases are typically found in women aged 25 to 61 years, with an average onset of 40 years [2]. They commonly have cystic architecture and are characterized by dilated cuboidal epithelium-lined ducts filled with mucin that can rupture and lead to the expulsion of acellular mucin into the periductal stroma, a key distinction from mucinous carcinoma. MLLs can occur anywhere in the breast, and the pathogenesis of MLLs is thought to be related to ductal obstruction from excessive mucinous secretion, but the exact mechanism remains unclear. These lesions range on a spectrum from benign to atypical to malignant. However, several reports have documented their high rate of developing dysplasia and association with atypical ductal hyperplasia (ADH), ductal carcinoma in situ (DCIS), and invasive carcinoma [3,4]. For these reasons, MLLs are being carefully considered as precursors to breast cancer, and surgical excision of such lesions is suggested due to their high rate of pre-malignant change.

Physical examination typically reveals a dome-shaped, non-tender, and mobile mass. MLLs are usually evaluated with mammography, ultrasonography, and core needle biopsy (CNB). Mammography of MLLs will show heterogeneously dense tissue with clustered round microcysts or pleomorphic calcifications [5]. Ultrasonography can reveal multiple oval-shaped cysts with solid areas with or without calcifications. Clustered cysts with thick septations and complex masses may also be found on ultrasound, associated with atypical proliferation or malignancy [6]. Radiologically, it is difficult to differentiate MLLs from other suspicious breast lesions; therefore, a core or excisional biopsy is needed for further evaluation and confirmation of the diagnosis. Myoepithelial cell markers [e.g., smooth muscle myosin heavy chain (SMMHC), calponin, p63] and positive staining patterns [e.g., mucicarmine, PAS diastase, Alcian blue] can be used to confirm the presence of myoepithelial cells in this condition and differentiate it from other breast conditions, such as cystic mastopathy, florid duct ectasia with luminal mucin, mucinous carcinoma, and nodular mucinosis. The presence of neovascularization in mucin seen on hematoxylin and eosin stain and immunohistochemistry has also been described as helpful in distinguishing mucinous carcinoma from MLLs [7].

## Case presentation

**Figure 1:**
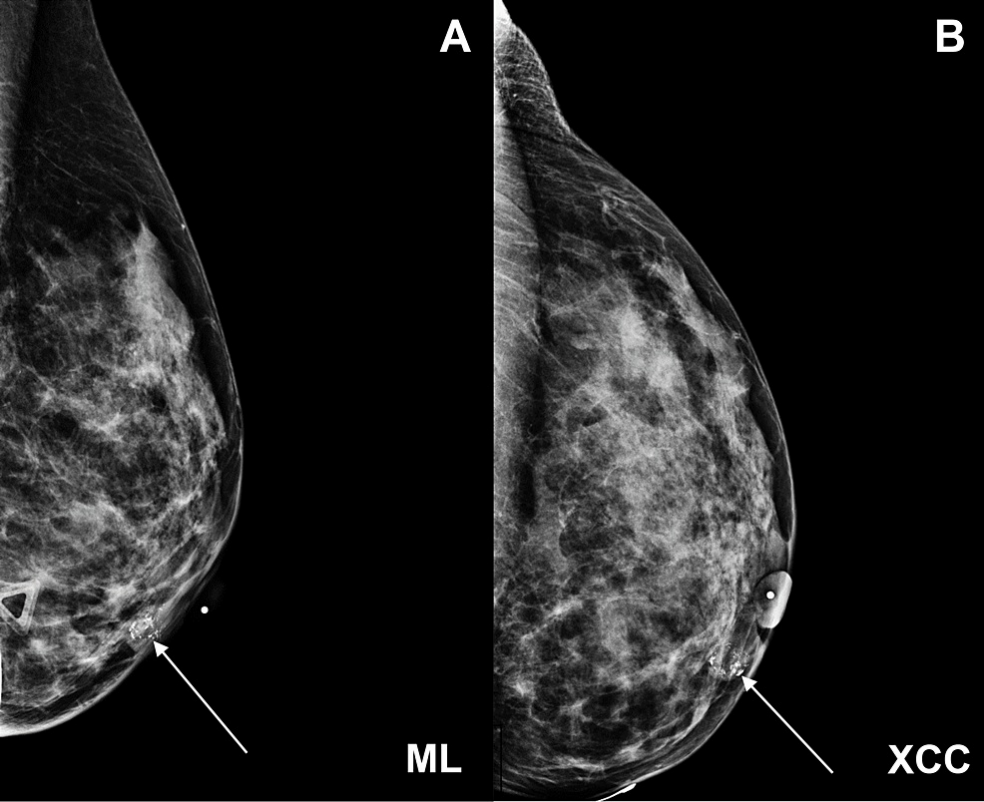
Mammography of the left breast obtained in (A) the mediolateral (ML) and (B) craniocaudal (XCC) views showed an 11 mm oval mass with circumscribed margins and coarse calcifications located at the central inner breast, anterior depth, 1 cm from the nipple.

**Figure 2:**
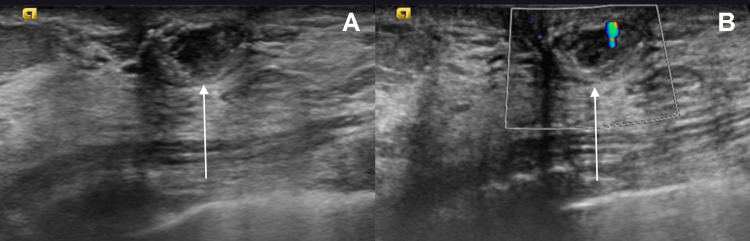
Ultrasonography of the left breast and axilla revealed a 10 x 10 x 6 mm solid mass with heterogeneous echotexture and coarse calcifications at 9 o’clock (A), 1 cm from the nipple. No internal color flow was appreciated on color Doppler imaging (B). The remainder of the breast and axilla ultrasound was unremarkable.

A 51-year-old woman was found to have an abnormality in her left breast on her initial screening mammogram. She did not report any symptoms associated with the abnormality. Physical examination revealed a mobile mass in her left breast without skin dimpling, nipple retraction, nipple discharge, peau d’orange, breast tenderness, or palpable lymphadenopathy. The patient denied any past breast surgery but did have a family history of breast cancer in her maternal great-aunt. Her mammogram showed an oval mass with circumscribed margins and coarse calcifications in the left breast (Figure 1). Ultrasound demonstrated the mass without any other sonographic abnormalities (Figure 2). The lesion was classified as Breast Imaging Reporting and Data System (BI-RADS) category 4A - low level of suspicion (>2% to <10% likelihood of malignancy), and a CNB was recommended.

**Figure 3:**
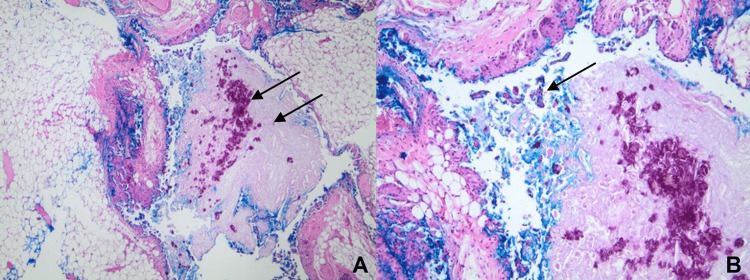
Histological findings of mucocele in the left breast lumpectomy. (A) Lumpectomy of the left breast shows a cyst filled with mucin and associated microcalcifications (40x magnification). (B) Myoepithelial cells adhere to strips of cells floating in lakes of mucin (100x magnification).

A CNB was performed, and the diagnosis of mucocele with mucinophage aggregate and calcifications in the mucin of benign breast tissue was made. A fiduciary was placed at the biopsy site, and resection of the high-risk lesion was recommended. Following a lumpectomy of the lesion in her left breast, the patient underwent surveillance without evidence of recurrence (Figure 3).

## Discussion

MLLs are rare and characterized by mucin-filled ducts or cysts rupturing the surrounding stroma. Due to their frequent atypia and malignant potential, excision of MLLs is typically performed following confirmation by CNB.

Calcifications in up to 93% of MLLs are often the primary radiological finding in screening mammography [8]. Several types of nonspecific calcifications have been reported in MLLs, which makes mammography alone difficult to establish a diagnosis [6]. However, calcifications may help determine the risk for malignancy. Towne et al. reported that all of their MLL cases contained widespread coarse calcifications that were upgraded following CNB, suggesting an association between coarse calcifications and higher upgrade rates [9]. Consistent with previous reports, our patient presented with coarse calcifications on her initial mammogram, and a follow-up ultrasound was recommended. Ultrasonographic evaluation of MLLs also shows nonspecific characteristics. The typical finding of low-echoic lesions resembling complex cysts and lack of flow on color Doppler is suggestive but not specific for MLLs. Additionally, the sonographic appearance of MLLs is often consistent with pure mucinous carcinomas, containing >90% mucin, and does not help distinguish between benign or malignant MLLs [10].

Since radiologic findings alone do not provide adequate evidence to diagnose MLLs, it raises the question of whether CNB can diagnose and determine the malignant potential of MLLs. The low cellularity of MLLs and small local tissue sampling from CNB reduce the likelihood of detection and diagnosis of MLLs. For these reasons, MLLs are believed to be underdiagnosed or misdiagnosed as other breast pathologies. Other diagnostic techniques, such as fine-needle aspiration cytology (FNAC), are less favored than CNB because of the smaller tissue sample and reduced structural information about the lesion. Reports of identical mucin makeup, similar cytological appearance, and difficult representative sampling of the whole lesion associated with FNAC of MLLs and mucinous carcinoma have led to the misdiagnosis of mucinous carcinoma in an MLL on at least one occasion [11]. There remains much uncertainty in diagnosing MLLs by CNB and FNAC, and the standard practice remains the excision of suspected lesions. These findings are consistent with Weaver et al.’s suggestion that MLLs and mucinous carcinoma exist on a pathological continuum and that surgical excision of all such lesions should be performed [12].

There needs to be more data in the literature regarding managing MLLs due to their rarity. However, more recent studies are proposing selective excision based on the histology of the lesion. One study found that MLLs with atypia were upgraded to DCIS twice as often as samples without atypia, occurring in approximately half of the biopsies [9]. Another study found that 28 out of the 53 MLL lesions analyzed were malignant, with 14 being in situ and the other 14 being invasive [13]. Comparatively, the upgrade rate of MLLs without atypia to invasive or in situ disease has been observed in less than three percent of cases [14]. ADH on histology also raises concern for the future development of intraductal carcinoma. As previously mentioned, the detection of atypia remains a challenge due to the low cellularity, localized biopsy, and intra-lesional heterogeneity of MLLs. The discovery of smaller malignancies in MLLs is often incidental and unlikely to be found on percutaneous biopsy. Therefore, the excisional biopsy of all MLL lesions found on CNB is still recommended for management [10].

Following the excision of a suspected MLL, surveillance consists of reassessment at six-month intervals for two to three years, then annually. This surveillance protocol is recommended for women who undergo excision and those who defer. Patients with ADH may choose to start chemoprevention with tamoxifen [1].

## Conclusions

Imaging is nonspecific for MLLs, but in this specific case, the mammogram demonstrated a superficial oval mass with coarse calcifications, and the breast ultrasound demonstrated a mass with heterogenous echotexture. Although the initial histologic evaluation of MLLs on CNB may appear benign, malignant components may also be present. A patient diagnosed with MLL by CNB should follow up with a breast surgeon for possible excisional biopsy for management. MLLs require a thorough evaluation of malignancy because of the overlapping radiographic and histologic findings of MLLs with other mucinous lesions. Further studies of MLLs can help improve the management of these lesions and prevent misdiagnosis and underdetection.

## References

[1] Ha D, Dialani V, Mehta TS, Keefe W, Iuanow E, Slanetz PJ (2015). Mucocele-like lesions in the breast diagnosed with percutaneous biopsy: is surgical excision necessary?. AJR Am J Roentgenol.

[2] Rosen PP (1986). Mucocele-like tumors of the breast. Am J Surg Pathol.

[3] Sutton B, Davion S, Feldman M, Siziopikou K, Mendelson E, Sullivan M (2012). Mucocele-like lesions diagnosed on breast core biopsy: assessment of upgrade rate and need for surgical excision. Am J Clin Pathol.

[4] Rakha EA, Shaaban AM, Haider SA (2013). Outcome of pure mucocele-like lesions diagnosed on breast core biopsy. Histopathology.

[5] Kim JY, Han BK, Choe YH, Ko YH (2005). Benign and malignant mucocele-like tumors of the breast: mammographic and sonographic appearances. AJR Am J Roentgenol.

[6] Kim SM, Kim HH, Kang DK, Shin HJ, Cho N, Park JM, Cha JH (2011). Mucocele-like tumors of the breast as cystic lesions: sonographic-pathologic correlation. AJR Am J Roentgenol.

[7] Onken AM, Collins LC, Schnitt SJ (2022). Mucin neovascularization as a diagnostic aid to distinguish mucinous carcinomas from mucocele-like lesions in breast and core needle biopsies. Am J Surg Pathol.

[8] Griffiths R, Alarcon L, Bonello V, Scott V, Szollosi Z (2021). Mucocele-like lesions of the breast - a radiological and clinicopathological analysis. Curr Probl Cancer.

[9] Towne WS, Michaels AY, Ginter PS (2022). Mucocele-like lesion of the breast diagnosed on core biopsy: histologic and clinical analysis of 78 cases with focus on features associated with upgrade. Arch Pathol Lab Med.

[10] Glazebrook K, Reynolds C (2003). Mucocele-like tumors of the breast: mammographic and sonographic appearances. AJR Am J Roentgenol.

[11] Kikuchi S, Nishimura R, Osako T, Okumura Y, Hayashi M, Toyozumi Y, Arima N (2012). Mucocele-like tumor associated with ductal carcinoma in situ diagnosed as mucinous carcinoma by fine-needle aspiration cytology: report of a case. Surg Today.

[12] Weaver MG, Abdul-Karim FW, Al-Kaisi N (1993). Mucinous lesions of the breast. a pathological continuum. Pathol Res Pract.

[13] Hamele-Bena D, Cranor ML, Rosen PP (1996). Mammary mucocele-like lesions. benign and malignant. Am J Surg Pathol.

[14] Gibreel WO, Boughey JC (2016). Mucocele-like lesions of the breast: rate of upstaging and cancer development. Ann Surg Oncol.

